# Bis[μ_2_-bis­(diphenyl­phosphan­yl)methane-κ^2^ *P*:*P*′]bis­(μ_4_-diphenyl­phosphinato-κ^4^ *O*:*O*:*O*′:*O*′)bis­(μ_2_-trifluoro­acetato-κ^2^ *O*:*O*′)tetra­silver(I) acetonitrile disolvate

**DOI:** 10.1107/S1600536811045466

**Published:** 2011-11-05

**Authors:** Li-Li Huang, Chen Jia, Li-Ping Tang, Bai Jing, Qi-Hua Deng

**Affiliations:** a Sichuan College of Chemical Technology, Luzhou 646005, People’s Republic of China; b School of Chemistry and Chemical Engineering, Guangxi Normal University, Guilin 541004, People’s Republic of China

## Abstract

In the cation of the title compound, [Ag_4_(C_2_F_3_O_2_)_2_(C_12_H_10_O_2_P)_2_(C_25_H_22_P_2_)_2_]·2CH_3_CN, the two independent Ag^+^ cations are four-coordinated in a distorted tetra­hedral geometry by one P atom from a bis­(diphenyl­phosphan­yl)methane (dppm) ligand, one O atom from a trifluoro­acetate anion and two O atoms from two diphenyl­phosphinate (dpp) ligands. Two dppm ligands, two dpp ligands and two trifluoro­acetate anions bridge four metal atoms, forming a centrosymmetric tetra­nuclear complex. Intra­molecular C—H⋯O hydrogen bonds and a weak π–π inter­action [centroid–centroid distance = 3.9804 (13) Å] are also observed.

## Related literature

For applications of metals complexes with diphosphine ligands, see: Catalano & Malwitz (2004 ▶); Chiu & Lee (2005 ▶). For related structures, see: Kuang *et al.* (2002 ▶); Rudler *et al.* (1997 ▶); Zank *et al.* (1999 ▶).
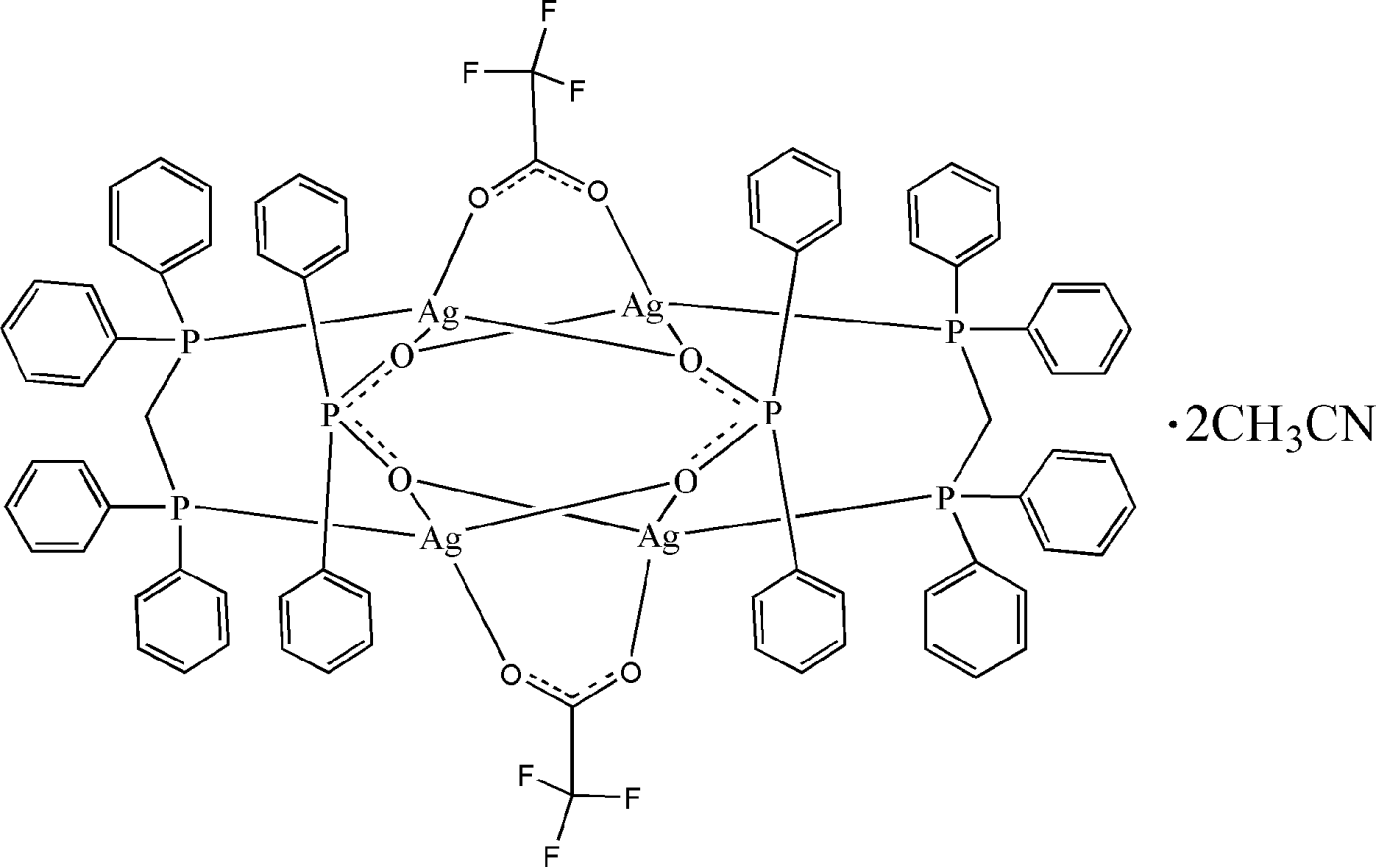


## Experimental

### 

#### Crystal data

[Ag_4_(C_2_F_3_O_2_)_2_(C_12_H_10_O_2_P)_2_(C_25_H_22_P_2_)_2_]·2C_2_H_3_N
*M* *_r_* = 1942.70
Monoclinic, 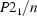

*a* = 11.724 (5) Å
*b* = 14.777 (6) Å
*c* = 24.183 (10) Å
β = 102.254 (5)°
*V* = 4094 (3) Å^3^
*Z* = 2
Mo *K*α radiation
μ = 1.13 mm^−1^
*T* = 296 K
0.20 × 0.20 × 0.20 mm

#### Data collection

Rigaku Mercury CCD diffractometer
Absorption correction: multi-scan (*CrystalClear*-SM Expert; Rigaku, 2009 ▶) *T*_min_ = 0.806, *T*_max_ = 0.806
34844 measured reflections
7192 independent reflections
6033 reflections with *I* > 2σ(*I*)
*R*_int_ = 0.057

#### Refinement

*R*[*F* ^2^ > 2σ(*F* ^2^)] = 0.046
*wR*(*F* ^2^) = 0.117
*S* = 1.01
7192 reflections
480 parameters
60 restraints
H-atom parameters constrained
Δρ_max_ = 1.08 e Å^−3^
Δρ_min_ = −0.59 e Å^−3^

### 

Data collection: *CrystalClear-SM Expert* (Rigaku, 2009 ▶); cell refinement: *CrystalClear-SM Expert*; data reduction: *CrystalClear-SM Expert*; program(s) used to solve structure: *SHELXS97* (Sheldrick, 2008 ▶); program(s) used to refine structure: *SHELXL97* (Sheldrick, 2008 ▶); molecular graphics: *SHELXTL* (Sheldrick, 2008 ▶); software used to prepare material for publication: *SHELXTL*.

## Supplementary Material

Crystal structure: contains datablock(s) I, global. DOI: 10.1107/S1600536811045466/rz2658sup1.cif

Structure factors: contains datablock(s) I. DOI: 10.1107/S1600536811045466/rz2658Isup2.hkl

Additional supplementary materials:  crystallographic information; 3D view; checkCIF report

## Figures and Tables

**Table 1:** Hydrogen-bond geometry (Å, °)

*D*—H⋯*A*	*D*—H	H⋯*A*	*D*⋯*A*	*D*—H⋯*A*
C19—H19⋯O3	0.93	2.46	3.380 (8)	169
C7—H7⋯O4^i^	0.93	2.45	3.368 (7)	171

Symmetry code: (i) 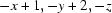.

**Fig. 1.**
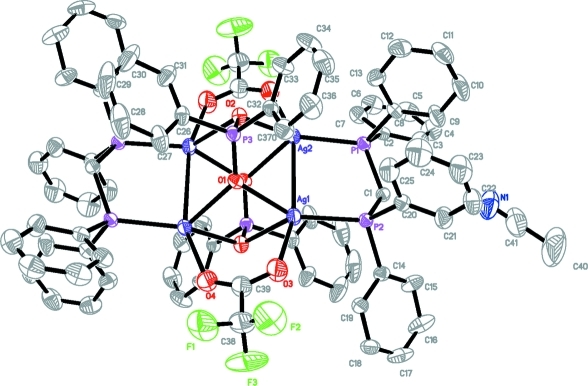
The molecular structure of title complex with displacement ellipsoids drawn at 50% probability level. Unlabelled atoms are related to the labelled atom by the symmetry operation 1-x, 2-y, -z. Hydrogen atoms are omitted for clarity.

## supplementary crystallographic information

### Comment

Over the past three decades, metals complexes containing diphosphine ligands attract great interest for their importance in their potential applications in dye-sensitized solar cells and photochemical catalysts (Catalano & Malwitz, 2004; Chiu & Lee, 2005). A series of silver(I) complexes containing diphosphine ligands have been synthesized (Kuang *et al.*, 2002). Synthetic tetranuclear silver(I) compounds have been widely reported (Rudler *et al.*, 1997; Zank *et al.*, 1999).

In the title compound (Fig. 1), the silver(I) atom adopts a distorted tetrahedral geometry through one P atom from a bis(diphenylphosphanyl)methane (dppm)ligand, one O atom from a trifluoroacetato anion and two O atoms from two diphenylphosphinate (dpp) ligands. Two dppm ligands, two dpp ligands and two trifluoroacetato anions bridge four metal atoms forming a centrosymmetric tetranuclear complex where the shortest Ag···Ag separation is 3.3655 (12) Å. The Ag—P and Ag—O bond distances are in the range 2.3685 (14)–2.3734 (14) and 2.278 (3)–2.505 (3) (14) Å, respectively. The O—Ag—P angles and O—Ag—O bond angles range from 80.56 (11) to 92.43 (14)° and from 106.90 (11) to 150.88 (8)°, respectively. In the crystal, intramolecular C—H···O hydrogen bonds are present (Table 1). In addition, a weak intramolecular π–π interaction of 3.9804 (13) Å between the centroids of  the C8—C13 and C20—C25 phenyl rings is observed.

### Experimental

The synthesis of the title compound was carried out by reacting silver trifluoroacetato (0.044 g, 0.2 mmol), diphenylphosphinic acid (0.022 g, 0.1 mmol) and bis(diphenylphosphine)methane (0.038 g, 0.1 mmol). The resulting mixture was allowed to stir for 1 h at room temperature. Colourless crystals of the title compound were obtained in 6 days by slow diffusion of diethyl ether into the solution (yield 15%).

### Refinement

All hydrogen atoms were generated geometrically and refined using a riding model, with C—H = 0.93–0.96Å and with *U*_iso_(H) = 1.2 *U*_eq_(C) or 1.5 *U*_eq_(C) for methyl H atoms. The restraints SIMU and DELU were used to ensure similar geometries and displacement parameters of the Ag1, O3, C38, C39 and F1–F3 atoms. The displacement parameters of the oxygen atoms were restrained to be approximately isotropic by means of the instruction ISOR (tolerance 0.02). Five low-theta reflections were omitted from the data set.

### Crystal data

**Table:**

[Ag_4_(C_2_F_3_O_2_)_2_(C_12_H_10_O_2_P)_2_(C_25_H_22_P_2_)_2_]·2C_2_H_3_N	*F*(000) = 1944
*M**_r_* = 1942.70	*D*_x_ = 1.576 Mg m^−^^3^
Monoclinic, *P*2_1_/*n*	Mo *K*α radiation, λ = 0.71073 Å
Hall symbol: -P 2yn	Cell parameters from 9421 reflections
*a* = 11.724 (5) Å	θ = 2.1–27.5°
*b* = 14.777 (6) Å	µ = 1.13 mm^−^^1^
*c* = 24.183 (10) Å	*T* = 296 K
β = 102.254 (5)°	Prism, colourless
*V* = 4094 (3) Å^3^	0.20 × 0.20 × 0.20 mm
*Z* = 2	

### Data collection

**Table:**

Rigaku Mercury CCD diffractometer	7192 independent reflections
Radiation source: fine-focus sealed tube	6033 reflections with *I* > 2σ(*I*)
graphite	*R*_int_ = 0.057
Detector resolution: 13.6612 pixels mm^-1^	θ_max_ = 25.0°, θ_min_ = 2.5°
ω scans	*h* = −13→13
Absorption correction: multi-scan (*CrystalClear*-SM Expert; Rigaku, 2009)	*k* = −17→17
*T*_min_ = 0.806, *T*_max_ = 0.806	*l* = −28→28
34844 measured reflections	

### Refinement

**Table:**

Refinement on *F*^2^	Primary atom site location: structure-invariant direct methods
Least-squares matrix: full	Secondary atom site location: difference Fourier map
*R*[*F*^2^ > 2σ(*F*^2^)] = 0.046	Hydrogen site location: inferred from neighbouring sites
*wR*(*F*^2^) = 0.117	H-atom parameters constrained
*S* = 1.01	*w* = 1/[σ^2^(*F*_o_^2^) + (0.0503*P*)^2^ + 7.4307*P*] where *P* = (*F*_o_^2^ + 2*F*_c_^2^)/3
7192 reflections	(Δ/σ)_max_ = 0.001
480 parameters	Δρ_max_ = 1.08 e Å^−^^3^
60 restraints	Δρ_min_ = −0.59 e Å^−^^3^

### Special details

**Table:**

Geometry. All e.s.d.'s (except the e.s.d. in the dihedral angle between two l.s. planes) are estimated using the full covariance matrix. The cell e.s.d.'s are taken into account individually in the estimation of e.s.d.'s in distances, angles and torsion angles; correlations between e.s.d.'s in cell parameters are only used when they are defined by crystal symmetry. An approximate (isotropic) treatment of cell e.s.d.'s is used for estimating e.s.d.'s involving l.s. planes.
Refinement. Refinement of *F*^2^ against ALL reflections. The weighted *R*-factor *wR* and goodness of fit *S* are based on *F*^2^, conventional *R*-factors *R* are based on *F*, with *F* set to zero for negative *F*^2^. The threshold expression of *F*^2^ > σ(*F*^2^) is used only for calculating *R*-factors(gt) *etc*. and is not relevant to the choice of reflections for refinement. *R*-factors based on *F*^2^ are statistically about twice as large as those based on *F*, and *R*- factors based on ALL data will be even larger.

### Fractional atomic coordinates and isotropic or equivalent isotropic displacement parameters (Å^2^)

**Table:**

	*x*	*y*	*z*	*U*_iso_*/*U*_eq_	
Ag1	0.54210 (3)	0.92166 (3)	0.087708 (16)	0.04807 (13)	
Ag2	0.66296 (3)	0.91321 (2)	−0.026597 (15)	0.04551 (13)	
C1	0.6904 (4)	0.7175 (3)	0.06180 (18)	0.0384 (10)	
H1A	0.7435	0.6710	0.0803	0.046*	
H1B	0.6232	0.6873	0.0389	0.046*	
C2	0.7857 (4)	0.6966 (3)	−0.0368 (2)	0.0457 (11)	
C3	0.8256 (6)	0.6101 (4)	−0.0213 (3)	0.0708 (17)	
H3	0.8354	0.5916	0.0162	0.085*	
C4	0.8511 (7)	0.5508 (5)	−0.0616 (3)	0.087 (2)	
H4	0.8787	0.4930	−0.0508	0.104*	
C5	0.8363 (6)	0.5760 (5)	−0.1163 (3)	0.083 (2)	
H5	0.8561	0.5364	−0.1427	0.099*	
C6	0.7922 (6)	0.6594 (5)	−0.1331 (3)	0.0785 (19)	
H6	0.7787	0.6756	−0.1711	0.094*	
C7	0.7674 (5)	0.7208 (4)	−0.0931 (2)	0.0618 (14)	
H7	0.7384	0.7779	−0.1046	0.074*	
C8	0.9111 (4)	0.8015 (3)	0.0557 (2)	0.0438 (11)	
C9	0.9731 (5)	0.7357 (4)	0.0905 (3)	0.081 (2)	
H9	0.9382	0.6805	0.0951	0.098*	
C10	1.0871 (6)	0.7523 (5)	0.1185 (4)	0.106 (3)	
H10	1.1290	0.7078	0.1414	0.128*	
C11	1.1380 (5)	0.8335 (5)	0.1127 (3)	0.086 (2)	
H11	1.2150	0.8435	0.1312	0.103*	
C12	1.0781 (6)	0.8999 (5)	0.0803 (3)	0.0767 (18)	
H12	1.1129	0.9557	0.0772	0.092*	
C13	0.9638 (5)	0.8835 (4)	0.0518 (2)	0.0608 (14)	
H13	0.9223	0.9290	0.0296	0.073*	
C14	0.5474 (4)	0.7051 (3)	0.14380 (18)	0.0438 (11)	
C15	0.5558 (6)	0.6126 (4)	0.1397 (2)	0.0673 (16)	
H15	0.6127	0.5872	0.1230	0.081*	
C16	0.4787 (7)	0.5568 (5)	0.1607 (3)	0.087 (2)	
H16	0.4850	0.4942	0.1584	0.105*	
C17	0.3939 (6)	0.5940 (6)	0.1848 (3)	0.085 (2)	
H17	0.3410	0.5567	0.1976	0.102*	
C18	0.3867 (5)	0.6852 (6)	0.1902 (3)	0.075 (2)	
H18	0.3307	0.7098	0.2079	0.090*	
C19	0.4623 (4)	0.7420 (4)	0.1696 (2)	0.0590 (14)	
H19	0.4563	0.8044	0.1729	0.071*	
C20	0.7694 (4)	0.7932 (3)	0.17396 (19)	0.0448 (11)	
C21	0.8085 (5)	0.7232 (4)	0.2106 (3)	0.0722 (17)	
H21	0.7679	0.6687	0.2071	0.087*	
C22	0.9087 (6)	0.7338 (6)	0.2529 (3)	0.091 (2)	
H22	0.9336	0.6863	0.2778	0.109*	
C23	0.9698 (7)	0.8112 (6)	0.2584 (3)	0.100 (3)	
H23	1.0357	0.8174	0.2873	0.120*	
C24	0.9360 (7)	0.8798 (6)	0.2221 (4)	0.106 (3)	
H24	0.9804	0.9324	0.2252	0.128*	
C25	0.8344 (6)	0.8724 (4)	0.1798 (3)	0.0770 (18)	
H25	0.8103	0.9208	0.1556	0.092*	
C26	0.6109 (4)	1.2297 (3)	0.0284 (2)	0.0469 (12)	
C27	0.5551 (6)	1.2773 (4)	0.0637 (3)	0.0758 (19)	
H27	0.5217	1.2470	0.0900	0.091*	
C28	0.5490 (8)	1.3714 (6)	0.0598 (4)	0.113 (3)	
H28	0.5077	1.4036	0.0821	0.135*	
C29	0.6024 (9)	1.4159 (5)	0.0238 (5)	0.126 (5)	
H29	0.6035	1.4788	0.0242	0.152*	
C30	0.6548 (8)	1.3706 (5)	−0.0131 (4)	0.109 (3)	
H30	0.6870	1.4023	−0.0393	0.131*	
C31	0.6599 (5)	1.2760 (4)	−0.0113 (3)	0.0732 (18)	
H31	0.6955	1.2443	−0.0362	0.088*	
C32	0.7743 (4)	1.1088 (3)	0.08724 (19)	0.0411 (10)	
C33	0.8771 (4)	1.1165 (4)	0.0682 (3)	0.0623 (15)	
H33	0.8753	1.1158	0.0295	0.075*	
C34	0.9828 (5)	1.1255 (5)	0.1063 (3)	0.083 (2)	
H34	1.0516	1.1303	0.0932	0.100*	
C35	0.9864 (6)	1.1272 (5)	0.1625 (3)	0.083 (2)	
H35	1.0577	1.1337	0.1878	0.099*	
C36	0.8866 (6)	1.1196 (5)	0.1822 (3)	0.081 (2)	
H36	0.8901	1.1208	0.2210	0.097*	
C38	0.3389 (7)	1.0357 (6)	0.2187 (3)	0.0934 (15)	
C39	0.3725 (6)	1.0221 (5)	0.1619 (3)	0.0804 (12)	
C40	0.8585 (15)	0.3690 (11)	0.1793 (11)	0.393 (18)	
H40A	0.9376	0.3619	0.1999	0.590*	
H40B	0.8366	0.3177	0.1551	0.590*	
H40C	0.8076	0.3734	0.2055	0.590*	
C41	0.8488 (9)	0.4537 (7)	0.1442 (6)	0.150 (5)	
C37O	0.7795 (3)	1.1099 (2)	0.14489 (10)	0.0608 (14)	
H37O	0.7116	1.1042	0.1587	0.073*	
F1	0.2990 (3)	1.1197 (2)	0.22513 (10)	0.1335 (18)	
F2	0.4197 (3)	1.0317 (2)	0.26317 (10)	0.155 (2)	
F3	0.2555 (3)	0.9843 (2)	0.22562 (10)	0.160 (2)	
N1	0.8428 (7)	0.5182 (5)	0.1215 (4)	0.124 (3)	
O1	0.5435 (3)	1.0716 (2)	0.06592 (14)	0.0466 (8)	
O2	0.6484 (3)	1.0685 (2)	−0.01761 (13)	0.0440 (7)	
O3	0.4545 (4)	0.9706 (3)	0.16520 (18)	0.0879 (12)	
O4	0.3134 (4)	1.0629 (3)	0.12293 (16)	0.0762 (12)	
P1	0.76367 (10)	0.78230 (8)	0.01470 (5)	0.0374 (3)	
P2	0.64141 (10)	0.78438 (8)	0.11642 (5)	0.0375 (3)	
P3	0.63580 (10)	1.10991 (8)	0.03764 (5)	0.0385 (3)	

### Atomic displacement parameters (Å^2^)

**Table:**

	*U*^11^	*U*^22^	*U*^33^	*U*^12^	*U*^13^	*U*^23^
Ag1	0.0513 (2)	0.0492 (2)	0.0450 (2)	0.01425 (17)	0.01323 (17)	0.00685 (16)
Ag2	0.0429 (2)	0.0487 (2)	0.0454 (2)	0.01025 (16)	0.01052 (16)	0.00678 (15)
C1	0.039 (2)	0.041 (2)	0.036 (2)	0.0013 (19)	0.0084 (18)	0.0010 (18)
C2	0.040 (2)	0.051 (3)	0.050 (3)	−0.003 (2)	0.016 (2)	−0.005 (2)
C3	0.100 (5)	0.055 (3)	0.065 (4)	0.013 (3)	0.033 (3)	−0.006 (3)
C4	0.111 (6)	0.063 (4)	0.094 (5)	0.008 (4)	0.041 (4)	−0.023 (4)
C5	0.084 (5)	0.090 (5)	0.080 (5)	−0.007 (4)	0.029 (4)	−0.042 (4)
C6	0.086 (5)	0.103 (5)	0.049 (3)	−0.006 (4)	0.022 (3)	−0.024 (3)
C7	0.060 (3)	0.076 (4)	0.052 (3)	0.003 (3)	0.016 (3)	−0.010 (3)
C8	0.033 (2)	0.050 (3)	0.048 (3)	0.004 (2)	0.009 (2)	−0.005 (2)
C9	0.046 (3)	0.058 (4)	0.127 (6)	0.003 (3)	−0.012 (3)	0.002 (4)
C10	0.052 (4)	0.079 (5)	0.162 (8)	0.008 (4)	−0.035 (4)	0.005 (5)
C11	0.043 (3)	0.095 (5)	0.109 (6)	−0.002 (4)	−0.006 (3)	−0.021 (4)
C12	0.065 (4)	0.079 (4)	0.083 (5)	−0.028 (4)	0.009 (3)	−0.009 (4)
C13	0.059 (3)	0.062 (3)	0.059 (3)	−0.009 (3)	0.008 (3)	0.007 (3)
C14	0.042 (2)	0.056 (3)	0.033 (2)	−0.004 (2)	0.0079 (19)	0.003 (2)
C15	0.094 (5)	0.064 (4)	0.050 (3)	−0.018 (3)	0.030 (3)	−0.001 (3)
C16	0.134 (7)	0.074 (4)	0.062 (4)	−0.047 (4)	0.036 (4)	−0.004 (3)
C17	0.081 (5)	0.122 (6)	0.054 (4)	−0.048 (5)	0.019 (3)	0.013 (4)
C18	0.043 (3)	0.122 (6)	0.063 (4)	0.004 (4)	0.017 (3)	0.036 (4)
C19	0.048 (3)	0.079 (4)	0.052 (3)	0.009 (3)	0.014 (2)	0.022 (3)
C20	0.043 (3)	0.054 (3)	0.035 (2)	−0.001 (2)	0.0044 (19)	−0.002 (2)
C21	0.063 (4)	0.074 (4)	0.071 (4)	0.002 (3)	−0.005 (3)	0.010 (3)
C22	0.072 (4)	0.116 (6)	0.070 (4)	0.013 (4)	−0.017 (3)	0.020 (4)
C23	0.084 (5)	0.113 (6)	0.083 (5)	−0.004 (5)	−0.028 (4)	−0.005 (5)
C24	0.093 (6)	0.102 (6)	0.105 (6)	−0.029 (5)	−0.024 (5)	−0.024 (5)
C25	0.076 (4)	0.062 (4)	0.082 (4)	−0.001 (3)	−0.007 (3)	−0.007 (3)
C26	0.044 (3)	0.042 (3)	0.046 (3)	0.003 (2)	−0.009 (2)	−0.003 (2)
C27	0.074 (4)	0.064 (4)	0.080 (4)	0.024 (3)	−0.006 (3)	−0.022 (3)
C28	0.115 (7)	0.074 (5)	0.125 (8)	0.035 (5)	−0.030 (6)	−0.045 (5)
C29	0.131 (9)	0.043 (4)	0.162 (11)	0.012 (5)	−0.065 (8)	−0.015 (5)
C30	0.119 (7)	0.060 (5)	0.126 (7)	−0.023 (5)	−0.027 (6)	0.037 (5)
C31	0.072 (4)	0.053 (3)	0.083 (4)	−0.014 (3)	−0.010 (3)	0.017 (3)
C32	0.038 (2)	0.041 (2)	0.043 (3)	−0.001 (2)	0.0029 (19)	−0.0015 (19)
C33	0.041 (3)	0.086 (4)	0.061 (3)	−0.007 (3)	0.011 (2)	−0.008 (3)
C34	0.035 (3)	0.117 (6)	0.094 (5)	−0.010 (3)	0.008 (3)	−0.013 (4)
C35	0.051 (4)	0.101 (5)	0.080 (5)	0.001 (4)	−0.020 (3)	−0.009 (4)
C36	0.075 (4)	0.107 (5)	0.049 (3)	0.004 (4)	−0.015 (3)	−0.002 (3)
C38	0.108 (3)	0.123 (3)	0.062 (3)	0.014 (3)	0.047 (2)	0.003 (3)
C39	0.099 (3)	0.096 (3)	0.060 (2)	0.034 (2)	0.048 (2)	0.013 (2)
C40	0.195 (16)	0.238 (18)	0.68 (4)	−0.046 (14)	−0.05 (2)	0.32 (2)
C41	0.095 (7)	0.094 (7)	0.238 (14)	−0.001 (6)	−0.019 (8)	0.025 (8)
C37O	0.055 (3)	0.075 (4)	0.050 (3)	0.002 (3)	0.004 (2)	0.001 (3)
F1	0.157 (4)	0.148 (4)	0.111 (3)	0.031 (3)	0.061 (3)	−0.034 (3)
F2	0.164 (5)	0.242 (6)	0.056 (3)	0.039 (5)	0.018 (3)	0.006 (3)
F3	0.210 (5)	0.187 (5)	0.121 (4)	−0.077 (4)	0.121 (4)	−0.039 (3)
N1	0.138 (7)	0.076 (5)	0.142 (7)	0.013 (5)	−0.005 (5)	0.022 (4)
O1	0.0314 (16)	0.0489 (19)	0.059 (2)	−0.0002 (14)	0.0086 (14)	0.0036 (15)
O2	0.0459 (18)	0.0400 (17)	0.0433 (18)	0.0018 (14)	0.0030 (14)	−0.0048 (13)
O3	0.108 (3)	0.101 (3)	0.067 (2)	0.042 (2)	0.0471 (19)	0.013 (2)
O4	0.085 (3)	0.103 (3)	0.047 (2)	0.024 (3)	0.027 (2)	0.019 (2)
P1	0.0333 (6)	0.0411 (6)	0.0386 (6)	0.0049 (5)	0.0098 (5)	0.0001 (5)
P2	0.0355 (6)	0.0428 (6)	0.0348 (6)	0.0044 (5)	0.0086 (5)	0.0029 (5)
P3	0.0337 (6)	0.0376 (6)	0.0416 (6)	−0.0001 (5)	0.0021 (5)	−0.0008 (5)

### Geometric parameters (Å, °)

**Table:**

Ag1—O1	2.278 (3)	C20—P2	1.822 (5)
Ag1—P2	2.3685 (14)	C21—C22	1.393 (8)
Ag1—O3	2.430 (4)	C21—H21	0.9300
Ag1—O2^i^	2.505 (3)	C22—C23	1.342 (10)
Ag1—Ag2	3.3655 (12)	C22—H22	0.9300
Ag2—O2	2.314 (3)	C23—C24	1.343 (11)
Ag2—P1	2.3734 (14)	C23—H23	0.9300
Ag2—O1^i^	2.416 (3)	C24—C25	1.400 (9)
Ag2—O4^i^	2.429 (4)	C24—H24	0.9300
C1—P1	1.835 (4)	C25—H25	0.9300
C1—P2	1.837 (4)	C26—C27	1.375 (8)
C1—H1A	0.9700	C26—C31	1.396 (8)
C1—H1B	0.9700	C26—P3	1.800 (5)
C2—C7	1.380 (7)	C27—C28	1.394 (10)
C2—C3	1.386 (8)	C27—H27	0.9300
C2—P1	1.832 (5)	C28—C29	1.346 (14)
C3—C4	1.390 (8)	C28—H28	0.9300
C3—H3	0.9300	C29—C30	1.362 (14)
C4—C5	1.350 (10)	C29—H29	0.9300
C4—H4	0.9300	C30—C31	1.399 (9)
C5—C6	1.365 (10)	C30—H30	0.9300
C5—H5	0.9300	C31—H31	0.9300
C6—C7	1.399 (8)	C32—C37O	1.383 (5)
C6—H6	0.9300	C32—C33	1.384 (7)
C7—H7	0.9300	C32—P3	1.802 (5)
C8—C13	1.373 (7)	C33—C34	1.385 (8)
C8—C9	1.386 (8)	C33—H33	0.9300
C8—P1	1.823 (5)	C34—C35	1.350 (9)
C9—C10	1.386 (8)	C34—H34	0.9300
C9—H9	0.9300	C35—C36	1.359 (10)
C10—C11	1.360 (10)	C35—H35	0.9300
C10—H10	0.9300	C36—C37O	1.390 (7)
C11—C12	1.355 (9)	C36—H36	0.9300
C11—H11	0.9300	C38—F2	1.275 (8)
C12—C13	1.393 (8)	C38—F3	1.277 (9)
C12—H12	0.9300	C38—F1	1.347 (9)
C13—H13	0.9300	C38—C39	1.521 (8)
C14—C15	1.376 (7)	C39—O4	1.206 (7)
C14—C19	1.394 (7)	C39—O3	1.215 (7)
C14—P2	1.826 (5)	C40—C41	1.503 (16)
C15—C16	1.396 (8)	C40—H40A	0.9600
C15—H15	0.9300	C40—H40B	0.9600
C16—C17	1.370 (10)	C40—H40C	0.9600
C16—H16	0.9300	C41—N1	1.096 (11)
C17—C18	1.358 (10)	C37O—H37O	0.9300
C17—H17	0.9300	O1—P3	1.508 (3)
C18—C19	1.388 (8)	O1—Ag2^i^	2.416 (3)
C18—H18	0.9300	O2—P3	1.505 (3)
C19—H19	0.9300	O2—Ag1^i^	2.505 (3)
C20—C21	1.376 (7)	O4—Ag2^i^	2.429 (4)
C20—C25	1.387 (8)		
			
O1—Ag1—P2	150.88 (8)	C21—C22—H22	119.4
O1—Ag1—O3	85.06 (14)	C22—C23—C24	120.1 (7)
P2—Ag1—O3	106.90 (11)	C22—C23—H23	120.0
O1—Ag1—O2^i^	80.56 (11)	C24—C23—H23	120.0
P2—Ag1—O2^i^	124.08 (8)	C23—C24—C25	120.4 (7)
O3—Ag1—O2^i^	92.43 (14)	C23—C24—H24	119.8
O1—Ag1—Ag2	79.70 (9)	C25—C24—H24	119.8
P2—Ag1—Ag2	86.71 (3)	C20—C25—C24	120.2 (7)
O3—Ag1—Ag2	164.76 (12)	C20—C25—H25	119.9
O2^i^—Ag1—Ag2	85.22 (8)	C24—C25—H25	119.9
O2—Ag2—P1	143.96 (8)	C27—C26—C31	119.6 (6)
O2—Ag2—O1^i^	81.77 (11)	C27—C26—P3	120.7 (5)
P1—Ag2—O1^i^	127.51 (8)	C31—C26—P3	119.3 (4)
O2—Ag2—O4^i^	88.39 (14)	C26—C27—C28	119.5 (8)
P1—Ag2—O4^i^	111.96 (11)	C26—C27—H27	120.2
O1^i^—Ag2—O4^i^	85.26 (13)	C28—C27—H27	120.2
O2—Ag2—Ag1	80.43 (8)	C29—C28—C27	120.5 (9)
P1—Ag2—Ag1	86.74 (3)	C29—C28—H28	119.8
O1^i^—Ag2—Ag1	76.03 (8)	C27—C28—H28	119.8
O4^i^—Ag2—Ag1	159.35 (10)	C28—C29—C30	121.3 (8)
P1—C1—P2	115.1 (2)	C28—C29—H29	119.4
P1—C1—H1A	108.5	C30—C29—H29	119.4
P2—C1—H1A	108.5	C29—C30—C31	119.5 (9)
P1—C1—H1B	108.5	C29—C30—H30	120.2
P2—C1—H1B	108.5	C31—C30—H30	120.2
H1A—C1—H1B	107.5	C26—C31—C30	119.4 (8)
C7—C2—C3	118.6 (5)	C26—C31—H31	120.3
C7—C2—P1	118.5 (4)	C30—C31—H31	120.3
C3—C2—P1	122.8 (4)	C37O—C32—C33	118.6 (4)
C2—C3—C4	120.2 (6)	C37O—C32—P3	120.8 (3)
C2—C3—H3	119.9	C33—C32—P3	120.2 (4)
C4—C3—H3	119.9	C32—C33—C34	120.3 (6)
C5—C4—C3	120.8 (7)	C32—C33—H33	119.8
C5—C4—H4	119.6	C34—C33—H33	119.8
C3—C4—H4	119.6	C35—C34—C33	120.3 (6)
C4—C5—C6	120.1 (6)	C35—C34—H34	119.8
C4—C5—H5	119.9	C33—C34—H34	119.8
C6—C5—H5	119.9	C34—C35—C36	120.4 (6)
C5—C6—C7	120.1 (6)	C34—C35—H35	119.8
C5—C6—H6	119.9	C36—C35—H35	119.8
C7—C6—H6	119.9	C35—C36—C37O	120.5 (6)
C2—C7—C6	120.1 (6)	C35—C36—H36	119.8
C2—C7—H7	119.9	C37O—C36—H36	119.8
C6—C7—H7	119.9	F2—C38—F3	108.4 (6)
C13—C8—C9	118.4 (5)	F2—C38—F1	99.1 (6)
C13—C8—P1	119.2 (4)	F3—C38—F1	103.8 (5)
C9—C8—P1	122.4 (4)	F2—C38—C39	117.9 (6)
C10—C9—C8	120.0 (6)	F3—C38—C39	113.1 (7)
C10—C9—H9	120.0	F1—C38—C39	112.9 (6)
C8—C9—H9	120.0	O4—C39—O3	132.8 (6)
C11—C10—C9	120.3 (7)	O4—C39—C38	115.1 (6)
C11—C10—H10	119.9	O3—C39—C38	112.1 (6)
C9—C10—H10	119.9	C41—C40—H40A	109.5
C12—C11—C10	120.9 (6)	C41—C40—H40B	109.5
C12—C11—H11	119.5	H40A—C40—H40B	109.5
C10—C11—H11	119.5	C41—C40—H40C	109.5
C11—C12—C13	119.1 (6)	H40A—C40—H40C	109.5
C11—C12—H12	120.4	H40B—C40—H40C	109.5
C13—C12—H12	120.4	N1—C41—C40	175.8 (18)
C8—C13—C12	121.3 (6)	C32—C37O—C36	119.8 (4)
C8—C13—H13	119.4	C32—C37O—H37O	120.1
C12—C13—H13	119.4	C36—C37O—H37O	120.1
C15—C14—C19	119.3 (5)	P3—O1—Ag1	120.75 (18)
C15—C14—P2	123.7 (4)	P3—O1—Ag2^i^	122.82 (19)
C19—C14—P2	117.1 (4)	Ag1—O1—Ag2^i^	97.24 (11)
C14—C15—C16	120.0 (6)	P3—O2—Ag2	120.65 (18)
C14—C15—H15	120.0	P3—O2—Ag1^i^	109.62 (17)
C16—C15—H15	120.0	Ag2—O2—Ag1^i^	93.85 (11)
C17—C16—C15	120.1 (7)	C39—O3—Ag1	126.4 (4)
C17—C16—H16	119.9	C39—O4—Ag2^i^	131.2 (4)
C15—C16—H16	119.9	C8—P1—C2	102.9 (2)
C18—C17—C16	120.3 (6)	C8—P1—C1	105.0 (2)
C18—C17—H17	119.8	C2—P1—C1	102.3 (2)
C16—C17—H17	119.8	C8—P1—Ag2	115.56 (16)
C17—C18—C19	120.5 (6)	C2—P1—Ag2	113.99 (17)
C17—C18—H18	119.7	C1—P1—Ag2	115.47 (15)
C19—C18—H18	119.7	C20—P2—C14	103.4 (2)
C18—C19—C14	119.8 (6)	C20—P2—C1	104.8 (2)
C18—C19—H19	120.1	C14—P2—C1	102.7 (2)
C14—C19—H19	120.1	C20—P2—Ag1	115.88 (17)
C21—C20—C25	118.0 (5)	C14—P2—Ag1	111.04 (16)
C21—C20—P2	123.0 (4)	C1—P2—Ag1	117.35 (15)
C25—C20—P2	118.9 (4)	O2—P3—O1	117.7 (2)
C20—C21—C22	120.1 (6)	O2—P3—C26	109.4 (2)
C20—C21—H21	120.0	O1—P3—C26	108.3 (2)
C22—C21—H21	120.0	O2—P3—C32	110.1 (2)
C23—C22—C21	121.2 (7)	O1—P3—C32	109.1 (2)
C23—C22—H22	119.4	C26—P3—C32	101.0 (2)
			
O1—Ag1—Ag2—O2	4.02 (11)	O4—C39—O3—Ag1	−0.8 (14)
P2—Ag1—Ag2—O2	−150.12 (8)	C38—C39—O3—Ag1	178.5 (5)
O3—Ag1—Ag2—O2	3.6 (5)	O1—Ag1—O3—C39	48.8 (6)
O2^i^—Ag1—Ag2—O2	85.27 (11)	P2—Ag1—O3—C39	−158.3 (6)
O1—Ag1—Ag2—P1	150.22 (9)	O2^i^—Ag1—O3—C39	−31.5 (7)
P2—Ag1—Ag2—P1	−3.92 (4)	Ag2—Ag1—O3—C39	49.2 (10)
O3—Ag1—Ag2—P1	149.8 (5)	O3—C39—O4—Ag2^i^	−10.2 (14)
O2^i^—Ag1—Ag2—P1	−128.54 (8)	C38—C39—O4—Ag2^i^	170.4 (5)
O1—Ag1—Ag2—O1^i^	−79.80 (12)	C13—C8—P1—C2	112.4 (4)
P2—Ag1—Ag2—O1^i^	126.07 (8)	C9—C8—P1—C2	−66.9 (5)
O3—Ag1—Ag2—O1^i^	−80.2 (5)	C13—C8—P1—C1	−140.9 (4)
O2^i^—Ag1—Ag2—O1^i^	1.45 (10)	C9—C8—P1—C1	39.8 (5)
O1—Ag1—Ag2—O4^i^	−54.1 (3)	C13—C8—P1—Ag2	−12.5 (5)
P2—Ag1—Ag2—O4^i^	151.8 (3)	C9—C8—P1—Ag2	168.2 (4)
O3—Ag1—Ag2—O4^i^	−54.5 (6)	C7—C2—P1—C8	−112.0 (4)
O2^i^—Ag1—Ag2—O4^i^	27.1 (3)	C3—C2—P1—C8	64.6 (5)
C7—C2—C3—C4	2.7 (9)	C7—C2—P1—C1	139.2 (4)
P1—C2—C3—C4	−173.9 (5)	C3—C2—P1—C1	−44.1 (5)
C2—C3—C4—C5	−0.7 (11)	C7—C2—P1—Ag2	13.9 (4)
C3—C4—C5—C6	−2.1 (11)	C3—C2—P1—Ag2	−169.5 (4)
C4—C5—C6—C7	2.9 (11)	P2—C1—P1—C8	78.2 (3)
C3—C2—C7—C6	−1.9 (8)	P2—C1—P1—C2	−174.7 (2)
P1—C2—C7—C6	174.9 (5)	P2—C1—P1—Ag2	−50.3 (3)
C5—C6—C7—C2	−0.9 (10)	O2—Ag2—P1—C8	−26.8 (2)
C13—C8—C9—C10	−2.6 (10)	O1^i^—Ag2—P1—C8	−165.19 (19)
P1—C8—C9—C10	176.7 (6)	O4^i^—Ag2—P1—C8	93.4 (2)
C8—C9—C10—C11	1.0 (13)	Ag1—Ag2—P1—C8	−95.58 (17)
C9—C10—C11—C12	1.0 (13)	O2—Ag2—P1—C2	−145.7 (2)
C10—C11—C12—C13	−1.4 (12)	O1^i^—Ag2—P1—C2	75.89 (19)
C9—C8—C13—C12	2.2 (9)	O4^i^—Ag2—P1—C2	−25.5 (2)
P1—C8—C13—C12	−177.1 (5)	Ag1—Ag2—P1—C2	145.50 (16)
C11—C12—C13—C8	−0.3 (10)	O2—Ag2—P1—C1	96.2 (2)
C19—C14—C15—C16	0.7 (9)	O1^i^—Ag2—P1—C1	−42.2 (2)
P2—C14—C15—C16	−178.6 (5)	O4^i^—Ag2—P1—C1	−143.6 (2)
C14—C15—C16—C17	0.8 (10)	Ag1—Ag2—P1—C1	27.41 (16)
C15—C16—C17—C18	−2.3 (11)	C21—C20—P2—C14	31.1 (5)
C16—C17—C18—C19	2.4 (10)	C25—C20—P2—C14	−152.1 (5)
C17—C18—C19—C14	−0.9 (9)	C21—C20—P2—C1	−76.2 (5)
C15—C14—C19—C18	−0.6 (8)	C25—C20—P2—C1	100.6 (5)
P2—C14—C19—C18	178.7 (4)	C21—C20—P2—Ag1	152.8 (4)
C25—C20—C21—C22	1.6 (9)	C25—C20—P2—Ag1	−30.4 (5)
P2—C20—C21—C22	178.5 (5)	C15—C14—P2—C20	−85.6 (5)
C20—C21—C22—C23	−1.0 (12)	C19—C14—P2—C20	95.1 (4)
C21—C22—C23—C24	−1.2 (14)	C15—C14—P2—C1	23.2 (5)
C22—C23—C24—C25	2.5 (14)	C19—C14—P2—C1	−156.1 (4)
C21—C20—C25—C24	−0.3 (10)	C15—C14—P2—Ag1	149.5 (4)
P2—C20—C25—C24	−177.3 (6)	C19—C14—P2—Ag1	−29.8 (4)
C23—C24—C25—C20	−1.8 (13)	P1—C1—P2—C20	−84.1 (3)
C31—C26—C27—C28	−0.5 (9)	P1—C1—P2—C14	168.1 (2)
P3—C26—C27—C28	172.6 (5)	P1—C1—P2—Ag1	46.0 (3)
C26—C27—C28—C29	−3.5 (12)	O1—Ag1—P2—C20	43.1 (3)
C27—C28—C29—C30	5.9 (14)	O3—Ag1—P2—C20	−68.0 (2)
C28—C29—C30—C31	−4.1 (13)	O2^i^—Ag1—P2—C20	−173.02 (19)
C27—C26—C31—C30	2.2 (8)	Ag2—Ag1—P2—C20	105.01 (17)
P3—C26—C31—C30	−171.0 (5)	O1—Ag1—P2—C14	160.7 (2)
C29—C30—C31—C26	0.0 (11)	O3—Ag1—P2—C14	49.6 (2)
C37O—C32—C33—C34	−0.1 (9)	O2^i^—Ag1—P2—C14	−55.46 (19)
P3—C32—C33—C34	173.0 (5)	Ag2—Ag1—P2—C14	−137.43 (16)
C32—C33—C34—C35	−0.4 (11)	O1—Ag1—P2—C1	−81.6 (3)
C33—C34—C35—C36	0.5 (12)	O3—Ag1—P2—C1	167.3 (2)
C34—C35—C36—C37O	0.0 (12)	O2^i^—Ag1—P2—C1	62.24 (19)
F2—C38—C39—O4	−148.7 (7)	Ag2—Ag1—P2—C1	−19.73 (16)
F3—C38—C39—O4	83.5 (9)	Ag2—O2—P3—O1	−49.7 (3)
F1—C38—C39—O4	−34.0 (10)	Ag1^i^—O2—P3—O1	57.4 (2)
F2—C38—C39—O3	31.8 (11)	Ag2—O2—P3—C26	−173.8 (2)
F3—C38—C39—O3	−96.0 (8)	Ag1^i^—O2—P3—C26	−66.7 (2)
F1—C38—C39—O3	146.5 (7)	Ag2—O2—P3—C32	76.0 (2)
C33—C32—C37O—C36	0.6 (7)	Ag1^i^—O2—P3—C32	−176.91 (18)
P3—C32—C37O—C36	−172.6 (4)	Ag1—O1—P3—O2	56.4 (3)
C35—C36—C37O—C32	−0.5 (9)	Ag2^i^—O1—P3—O2	−67.5 (3)
P2—Ag1—O1—P3	33.9 (3)	Ag1—O1—P3—C26	−178.9 (2)
O3—Ag1—O1—P3	150.2 (2)	Ag2^i^—O1—P3—C26	57.2 (3)
O2^i^—Ag1—O1—P3	−116.5 (2)	Ag1—O1—P3—C32	−69.8 (3)
Ag2—Ag1—O1—P3	−29.65 (19)	Ag2^i^—O1—P3—C32	166.3 (2)
P2—Ag1—O1—Ag2^i^	169.19 (9)	C27—C26—P3—O2	155.7 (4)
O3—Ag1—O1—Ag2^i^	−74.42 (15)	C31—C26—P3—O2	−31.1 (5)
O2^i^—Ag1—O1—Ag2^i^	18.88 (12)	C27—C26—P3—O1	26.3 (5)
Ag2—Ag1—O1—Ag2^i^	105.69 (10)	C31—C26—P3—O1	−160.6 (4)
P1—Ag2—O2—P3	−50.7 (3)	C27—C26—P3—C32	−88.2 (4)
O1^i^—Ag2—O2—P3	97.1 (2)	C31—C26—P3—C32	84.9 (4)
O4^i^—Ag2—O2—P3	−177.4 (2)	C37O—C32—P3—O2	−155.2 (3)
Ag1—Ag2—O2—P3	20.03 (18)	C33—C32—P3—O2	31.7 (5)
P1—Ag2—O2—Ag1^i^	−166.27 (7)	C37O—C32—P3—O1	−24.8 (4)
O1^i^—Ag2—O2—Ag1^i^	−18.40 (11)	C33—C32—P3—O1	162.2 (4)
O4^i^—Ag2—O2—Ag1^i^	67.05 (14)	C37O—C32—P3—C26	89.1 (4)
Ag1—Ag2—O2—Ag1^i^	−95.51 (9)	C33—C32—P3—C26	−83.9 (5)

Symmetry codes: (i) −*x*+1, −*y*+2, −*z*.

### Hydrogen-bond geometry (Å, °)

**Table:**

*D*—H···*A*	*D*—H	H···*A*	*D*···*A*	*D*—H···*A*
C19—H19···O3	0.93	2.46	3.380 (8)	169
C7—H7···O4^i^	0.93	2.45	3.368 (7)	171

Symmetry codes: (i) −*x*+1, −*y*+2, −*z*.
